# Follow-up on pediatric patients with bronchiolitis obliterans treated with corticosteroid pulse therapy

**DOI:** 10.1186/s13023-014-0128-2

**Published:** 2014-08-15

**Authors:** Silvia Onoda Tomikawa, Fabíola Villac Adde, Luiz Vicente Ribeiro Ferreira da Silva Filho, Claudio Leone, Joaquim Carlos Rodrigues

**Affiliations:** Pediatric Pulmonology Division, Instituto da Criança, Hospital das Clínicas, University of São Paulo, Avenida Dr Enéas de Carvalho Aguiar, 647, CEP 05403-000 São Paulo, SP Brazil; Department of Maternal and Child Health, College of Public Health, University of São Paulo, Avenida Dr Arnaldo, 715, CEP 01246-904 São Paulo, SP Brazil; ᅟ, Rua Bianchi Bertoldi, 166 apt 101, CEP: 05422-070 São Paulo, SP Brazil

**Keywords:** Bronchiolitis obliterans, Pulse therapy, Methylprednisolone

## Abstract

**Background:**

Bronchiolitis obliterans (BO) is a rare but severe disease in children. Currently, there is no consensus on the treatment for BO with respect to the systemic use of corticosteroids. Here we report on the follow-up of children with a diagnosis of BO who were treated with corticosteroid pulse therapy.

**Methods:**

Forty patients fulfilling the BO diagnosis criteria were treated with methylprednisolone pulse therapy in monthly cycles until clinical improvement. After the pulse therapy began, we analyzed the clinical and laboratory data at intervals. Statistical analyses were performed using non-parametric tests to compare repeated measures (Friedman, Wilcoxon) or paired nominal data (McNemar) (α = 5%).

**Results:**

The frequency of wheezing exacerbations and hospitalizations was reduced (p = 0.0042 and p < 0.0001, respectively) and oxygen saturation improved (p = 0.0002) in the pulse therapy-treated patients. Prolonged oral corticosteroid therapy was discontinued in 83% of these patients. The mean Z-score length for age improved from -1.08 to -0.63, and the mean Z-score weight for age improved from -0.91 to -0.59. The adverse effects during the infusion were temporary and none were serious.

**Conclusions:**

Our data suggest that pulse corticotherapy could be a safe alternative to prolonged systemic oral corticotherapy in children with BO, thus minimizing the adverse effects of the oral therapy. New prospective controlled studies are required to confirm this proposition.

## Background

Bronchiolitis obliterans (BO) is a rare form of chronic obstructive lung disease that follows an infection-induced injury to the lower respiratory tract. This condition results in partial or complete obliteration of the small airways [1-3]. Although the first description of clinical cases of bronchiolitis obliterans is credited to Lange in 1901, many aspects of this disease remain unknown [4-6].

Bronchiolitis obliterans is an uncommon disease, but a growing number of reports in recent decades have resulted in a greater awareness of the condition [2,3]. Although there are no worldwide studies of the prevalence of post-infectious BO, it has mainly been reported in the southern hemisphere (Argentina, Chile, southern Brazil, Australia and New Zealand) [1,2,7].

No universally accepted protocol has been established for the treatment of BO [6,8]. Corticosteroid therapy seeks to modify the fibroblastic response in the early phase of illness. This approach is based on a study conducted in 1958 in which steroids were administered to rabbits to prevent development of BO [9]. Others studies have reported variable efficacy of corticosteroid therapy in patients with BO [1,5,10].

Methylprednisolone intravenous pulse therapy has been proposed to enhance the therapeutic effects and reduce the side effects of corticotherapy, and it is an alternative treatment for patients with more severe disease [1,7,10]. The aim of the present study was to review the clinical, laboratory and radiological data collected before and after treatment from patients with bronchiolitis obliterans who were treated with methylprednisolone pulse therapy.

## Methods

Forty children of both genders aged 5 months to 13 years and diagnosed with BO were followed up retrospectively at the Pediatric Pulmonology Unit of the Instituto da Criança, Hospital das Clínicas, University of São Paulo. The diagnosis of BO was made according to clinical criteria (acute lower respiratory tract infection associated with persistent obstructive respiratory disease after the initial event, which was not responsive to treatment for a period longer than 6 weeks), the results of high resolution computerized tomography of the thorax (mosaic pattern and/or bronchiectasis) and in some patients, with lung biopsies (obliterative bronchiolitis). Other causes of chronic obstructive pulmonary disease, including cystic fibrosis, bronchopulmonary dysplasia, pulmonary tuberculosis, α1-antitrypsin deficiency, immunodeficiencies, aspiration of foreign bodies and cardiac diseases, were excluded.

The cases included all patients who received at least six cycles of treatment with intravenous corticosteroid pulse therapy (methylprednisolone 30 mg/kg/BW per day for three days) from 1996 to 2007. The criteria for beginning corticosteroid pulse therapy in the patients with BO was the severity of the condition as indicated by difficulty in the removal of the prolonged oral corticosteroids and/or hypoxemia requiring home oxygen therapy.

This pulse regimen (30 mg/kg/day for 3 days) is already used in other childhood diseases such as renal and rheumatologic diseases [11-15]. The corticosteroid dose of 10 mg/kg/day is classically used in restrictive interstitial lung diseases, but we chose the 30 mg/kg/day dose due to the clinical severity of the disease in our patients. Recently, the 30 mg/kg/day pulse regimen has also been used in children with pneumonia caused by Mycoplasma pneumoniae [16].

The initial cycles of pulse therapy were repeated on a monthly basis. As clinical improvement was demonstrated, the cycle interval was increased to two months, and pulse therapy was later discontinued. All of the clinical and radiological data regarding the patients’ medical histories, such as duration of gestation, breast feeding, parental smoking and family history of asthma, were obtained from previous medical records.

### Statistical analysis

Clinical and laboratory data were analyzed at intervals after the pulse therapy began, and non-parametric statistical tests were used to compare repeated measures (Friedman, Wilcoxon) or paired nominal data (McNemar). A Dunn post-test with statistical significance defined as α = 5% was performed for multiple comparisons.

### Ethical approval

The ethics committee for analysis of clinical research projects of Hospital das Clinicas - University of São Paulo (CAPpesq) approved this research, protocol number 0695/07.

## Results

### Characteristics of the patients

Table 1 shows the characteristics and history of the 40 patients. Our study sample consisted of 30 (75%) boys and 10 (25%) girls, ranging in age (at diagnosis) from 5 months to 13 years. At diagnosis, the time elapsed since the episode of acute illness was between 1 and 134 months (mean 24.6 months, median 12.5 months). The average number of previous hospitalizations was 4 per year. Twenty-one patients (52.5%) required mechanical ventilation during a previous hospitalization, and 8 (20%) needed domiciliary oxygen therapy.

**Table 1:** **Characteristics and histories of 40 patients with bronchiolitis obliterans**

**Characteristics (n = 40)**			
Gender	Male = 30 (75%)	Female = 10 (25%)	
Gestational age	Full term = 35 (87.5%)	Premature = 5 (12.5%)	
Breast-feeding	<3 months = 21 (52.5%)	3-6 m = 17 (42.5%)	>6 months = 2 (5%)
Family history of asthma	28 (70%)		
Parental smoking	22 (55%)		
**History (n = 40)**			
Age at first wheezing	mean 7.4, median 4.5 (0-48 months)		
Age at onset of persistent wheezing	mean 18.3, median 9.5 (0-150 months)		
Age at diagnosis	mean 42.9, median 25 (5-156 months)		
Onset of disease/diagnosis interval	mean 24.6, median 12.5 (1-134 months)		
Recurrent pneumonia episodes	24 (60%)		
Hospitalization	mean 4 times per year, median 2.8 times per year (0-10 times per year)		
Mechanical ventilation assistance	21 (52.5%)		
Domiciliary oxygen therapy	8 (20%)		
Prolonged oral corticosteroid therapy	23 (57.5%)		
Inhaled corticosteroid therapy	19 (47.5%)		

Table 2 shows that the most common symptoms and signs of the 40 patients diagnosed with BO were wheezing, dyspnea and cough.

**Table 2:** **Symptoms and signs at time of diagnosis**

**Characteristics (n = 40)**	
**Symptoms**	
Persistent cough	23 (57.5%)
Dyspnea	26 (65%)
Persistent wheezing	40 (100%)
Cyanosis (reported episodes)	9 (22.5%)
**Physical examination**	
Increased anterior-posterior chest diameter	23 (57.5%)
Cushing syndrome-like aspect	8 (20%)
Clubbing fingers	7 (17.5%)
Watch glass nails	2 (5%)
Pulmonary auscultation	Diffuse crackles = 24 (60%)
	Localized crackles = 7 (17.5%)
	Wheezing = 33 (82.5%)

Table 3 shows that the most frequent findings of the chest HRCT were a mosaic perfusion pattern and bronchial wall thickening.

**Table 3:** HRCT* findings in children with bronchiolitis obliterans

**HRCT findings (n = 40)**	
Mosaic perfusion pattern	29 (72.5%)
Bronchial wall thickening	18 (45%)
Atelectasis	16 (40%)
Alveolar filling	12 (30%)
Bronchiectasis	11 (27.5%)
Hyperinflation	5 (12.5%)
Air trapping	5 (12.5%)
Swyer-James-MacLeod syndrome	1 (2.5%)

*HRTC- High resolution computed tomography.

### Etiology

The presumed etiology was post-infectious in 37 patients. Because viral identification for the acute bronchiolitis was not performed in all patients referred to the hospital, viral identification by paired serology was only available for 5 patients: 2 with adenovirus, 1 with respiratory syncytial virus (RSV), and 2 with adenovirus plus RSV. The other 32 patients had typical histories of previous severe acute bronchiolitis (most with hospitalization and even requiring mechanical ventilation) and after this event, the airway obstruction persisted for a period longer than 6 weeks. On this basis, these patients were considered to have post-infectious BO.

Two patients had histopathological findings consistent with obliterative bronchiolitis and indicative of aspiration syndrome. One patient had a history of acute viral bronchiolitis with persistence of airway obstruction and severe gastro-esophageal reflux. For this patient, we considered that viral infection and aspiration could be both associated with the etiology.

### Pathologic findings

Open lung biopsies were performed in 18 patients. In 14 of these, the pathologic findings were consistent with obliterative bronchiolitis. The histological patterns were: constrictive in 13 patients; proliferative in 1 patient; and inconclusive in 4 patients.

### Lung function measurements

Most patients did not perform a lung function test because they were under 6 years of age. Spirometry, including a bronchodilator response test, was performed in only 8 patients (ages 6-14, mean age 9.6 years). The means of the initial spirometry parameters, expressed as percentages of the predicted values, were: FVC: 77.2%; FEV_1_: 55.3%; FEV_1_/FVC: 67.4% and FEF_25-75%_: 35.7%. Three patients responded significantly to bronchodilation according to criteria from the American Thoracic Society and the European Respiratory Society [17].

Because patient follow-up was performed for many years, the first spirometry results were available for 24 patients (age 6-8 years, mean 7.2 years) after pulse therapy (mean time 27 months), with parameter means as follows: FVC: 66.8%; FEV_1_: 47.7%; FEV_1_/FVC: 65.6% and FEF_25-75%_: 26.6%. Five of these patients had a response to bronchodilation according the American Thoracic Society and the European Respiratory Society criteria [17].

### Follow-up

#### Clinical follow-up

The patients were followed for an average of 51.6 months (ranging from 23 to 93 months), and 24 patients are still being observed (Table 4). The total number of treatment cycles with pulse therapy ranged from 6 to 40 (mean 20.2, median 18.5).

**Table 4:** **Characteristics of the clinical follow-up of patients with BO**

**Characteristics (n = 40)**	
Age at beginning of follow-up	mean 40.9, median 25 (6-186 months)
Follow-up period	mean 51.6, median 49.2 (23-93 months)
Age at beginning of pulse therapy	mean 50.5, median 31.5 (6-180 months)
Onset of disease/pulse therapy interval	mean 32.1, median 18.5 (2-142 months)
Pulse therapy cycles	mean 20.2, median 18.5 (6-40 cycles)
Pulse therapy period	mean 26.2, median 24 (6-48 months)

In addition to the systemic corticosteroid therapy, all 40 patients received inhaled corticosteroids to reduce airway hyperreactivity for the entire follow-up period. Other medications used by the patients during the pulse therapy period included: long-term beta-2-agonist bronchodilators in 23 patients (57.5%); anti-gastroesophageal reflux drugs in 23 (57.5%); antibiotic prophylaxis in 19 (47.5%); anti-hypertensive drugs in 21 (52.5%); digoxin in 2 (5%); and diuretics in 13 (32.5%).

#### Development

**Figure 1:**
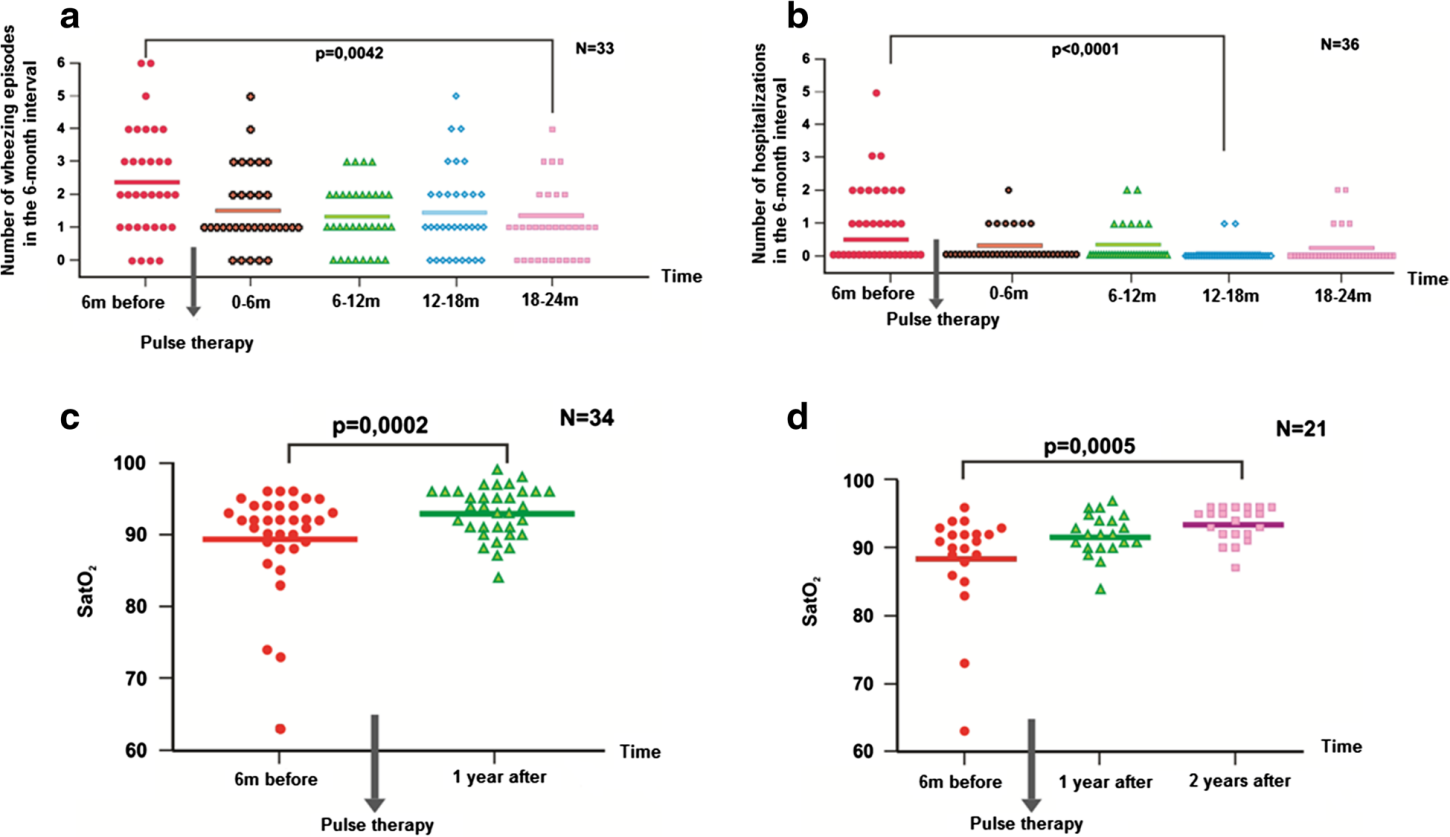
**Clinical and laboratory data before and after pulse therapy. a** – Wheezing exacerbations before and after pulse therapy (n = 33). **b** – Hospitalizations before and after pulse therapy (n = 36). **c** – Oxygen saturation (SatO_2_) before and after 1 year of pulse therapy (n = 34). **d** – Oxygen saturation (SatO_2_) before and after 1 and 2 years of pulse therapy (n = 21).

The clinical data were analyzed at intervals before and after pulse therapy began (every 6 months, before and after therapy). The frequency of the wheezing exacerbations (Figure 1a) was significantly reduced after 24 months of pulse therapy relative to the baseline (6 months before therapy) (p = 0.0042). The frequency of hospitalization after 18 months of pulse therapy (Figure 1b) was significantly reduced compared to the baseline (6 months before therapy) (p < 0.0001).

The initial transcutaneous oxygen saturation measurements (Figure 1c and d) showed hypoxemia (<95%) in 29 patients (n = 35). The level of oxygen saturation improved compared to the baseline in the first (p = 0.0002) and second years (p = 0.0005) following pulse therapy.

**Figure 2:**
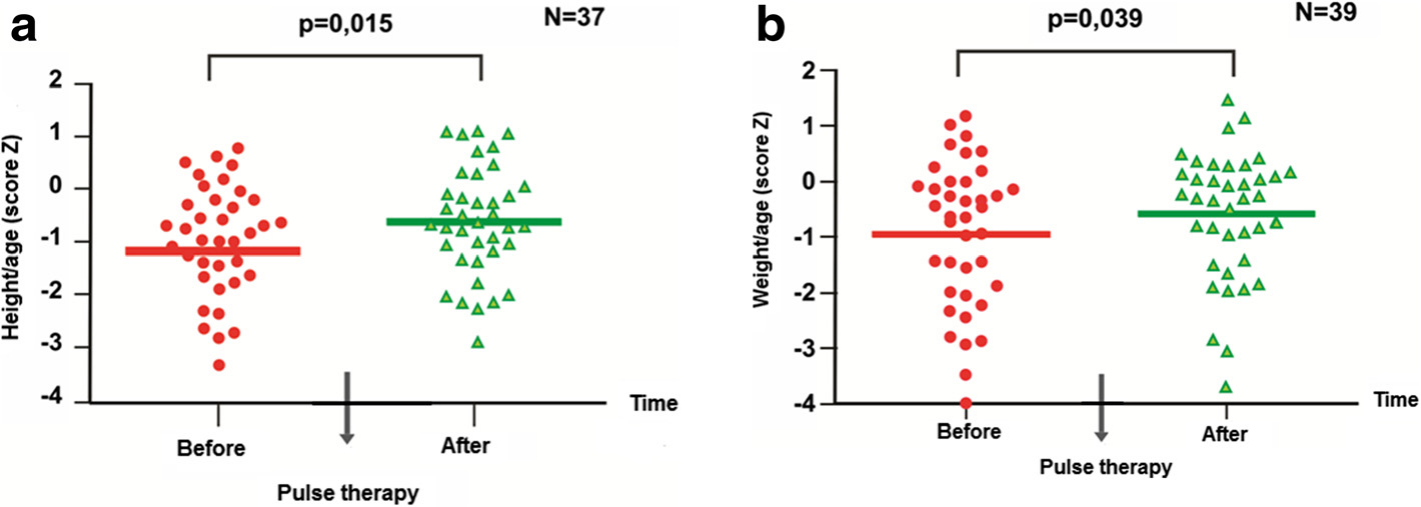
**Height and weight before and after pulse therapy. a **– Height-for-age (Z-score) before and after pulse therapy (n = 37). **b** – Weight-for-age (Z-score) before and after pulse therapy (n = 39).

The mean length-for-age Z-score (Figure 2a) improved compared to the baseline and after the end of pulse therapy, from -1.08 to -0.63 (p = 0.015). The mean weight-for-age Z-score (Figure 2b) was also improved at the end of pulse therapy compared to the baseline: from -0.91 to -0.59 (p = 0.039).

Spirometry was performed in 8 patients before pulse therapy. Only 5 patients repeated the exam after 1 year of treatment. The mean of the spirometric results 1 year before and 1 year after treatment were, respectively: FVC: 72.6%/78.9%; FEV_1_: 48.8%/52.6%; FEV_1_/CVF: 62.5%/63% and FEF_25-75%_: 24.2%/22.9%. Only an evaluation of the means is presented because it was not possible to perform a statistical analysis due the small number of patients.

During follow-up, chest tomography was repeated in 37 patients, and the initial abnormalities, such as bronchiectasis, bronchial wall thickening and mosaic perfusion patterns, remained in all of these patients. Normalization of tomographic alterations was not achieved during the follow-up period.

Before pulse therapy, 23 patients received continuous oral corticosteroid therapy. However, 24 months after starting pulse therapy, 19 patients (83%) were able to discontinue the oral corticosteroids at an average of 12.4 months (Table 5). The difference between the frequency of use of oral corticosteroids before and 24 months after pulse therapy is statistically significant (McNemar test, p <0.001).

**Table 5:** **Laboratory evaluation before and after pulse therapy**

**Characteristics (n = 40)**		
	**Before**	**After 24 m**
Cor pulmonale	27* (71%)	11** (47%)
Oral prolonged corticotherapy	23 (58%)	4 (10%)
Oxygen therapy		
Full-time	12 (30%)	4 (10%)
Overnight	8 (20%)	11 (28%)

*2 patients with no echocardiogram in their medical records.

**of 27 patients with an abnormal echocardiogram, 23 repeated the exam.

Prior to pulse therapy, 20 patients required supplemental oxygen therapy due to low oxygen saturation levels on room air and/or cor pulmonale (Table 5). The hypoxemia improved during follow up, and as a result, twenty-four months after starting the pulse therapy, the administration of oxygen therapy was totally discontinued in 5 patients (25%), while oxygen therapy was modified from full-time to overnight-only in another 5 patients (25%). The difference between the frequency of use of oxygen before and 24 months after pulse therapy is statistically significant (McNemar, p <0.029).

Echocardiograms showed evidence of pulmonary hypertension - mean pulmonary artery pressure above 25 mmHg at rest or above 30 mmHg in exercise, or systolic pulmonary artery pressure above 30 mmHg at rest or above 35 mmHg in exercise [18] - in 27 patients before pulse therapy. All patients with abnormal echocardiograms were referred to the Pediatric Cardiology Unit for evaluation. The cardiologist performed a physical exam with echocardiography and electrocardiography. Twenty-four months after starting pulse therapy, the cor pulmonale abnormalities disappeared in 12 patients (Table 5). The difference between the frequency of pulmonary hypertension before and 24 months after pulse therapy is statistically significant (McNemar, p <0.001).

### Comparison of patients with and without prolonged oral corticosteroids

Because 23 patients were already on continuous oral corticosteroid therapy at the beginning of the pulse therapy treatment and 17 were not, we compared the outcomes of the two groups.

**Figure 3:**
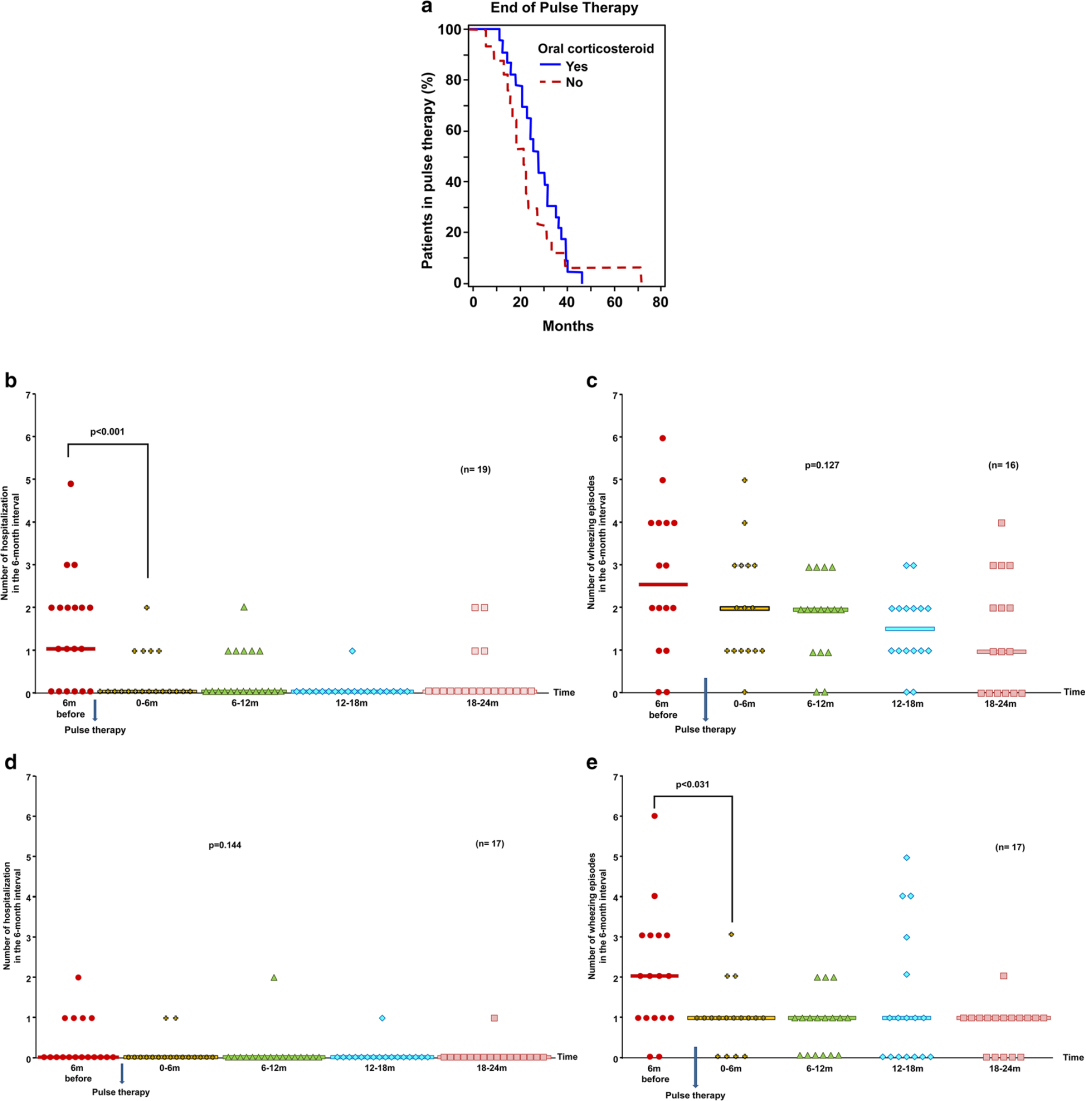
**Comparison of patients with and without prolonged oral corticosteroids. a **- Pulse therapy duration (months) in the group with and without prolonged oral corticosteroid therapy (n = 40). **b** - Hospitalizations before and after pulse therapy in the group with prolonged oral corticosteroid (n = 19). **c** - Wheezing exacerbations before and after pulse therapy in the group with prolonged oral corticosteroid (n = 16). **d** - Hospitalizations before and after pulse therapy in the group without prolonged oral corticosteroid (n = 17). **e** - Wheezing exacerbations before and after pulse therapy in the group without prolonged oral corticosteroid (n = 17).

Survival curves to compare the pulse duration of the patients who were on prolonged oral corticosteroid therapy and of the patients who were not given this therapy were produced (Figure 3a). The difference between the curves resulted not statistically significant (Log-rank test, p = 0.297).

Analysis of the frequency of wheezing exacerbations and hospitalizations showed a decrease in the number of hospitalizations in the group with prolonged oral corticosteroid therapy after pulse therapy compared to the baseline (6 months before therapy) (p < 0.001) (Figure 3b). Furthermore, the median number of hospitalizations before pulse therapy differed from the medians at all other times (6, 12, 18 and 24 months). However, no statistically significant differences among the median numbers of exacerbations were found (p = 0.127) (Figure 3c).

After pulse therapy, the group without prolonged oral corticosteroid therapy had a decrease in the number of exacerbations when compared to the baseline (6 month before therapy) (p < 0.031) (Figure 3e). Additionally, the median number of exacerbations before pulse therapy differed from the medians at all other times (6, 12, 18 and 24 months). However, no statistically significant differences among the median numbers of hospitalizations were found (p = 0.144) (Figure 3d).

### Adverse effects

#### Adverse effects during the infusion

All of the adverse effects during methylprednisolone intravenous infusion were temporary (Table 6). The most common adverse effect was hyperglycemia (9.1% of cycles), but none of these patients developed diabetes mellitus. Of the 19 episodes of acute hypertension, 10 occurred in patients with a prior diagnosis of systemic hypertension.

**Table 6:** **Adverse effects of pulse therapy**

**Adverse effects (number of cycles = 808)**	
Hyperglycemia > 200 mg/dl*	74 episodes (9% of cycles)
Hypernatremia	14 episodes (1.7% of cycles)
Acute hypertension	19 episodes (2.3% of cycles)

*patients did not fast because the visits to the day hospital were after lunch time.

#### Adverse effects of prolonged corticosteroid therapy

Bone mineral density was measured in 25 patients. The results were normal in 13, abnormal in 11 and borderline in 1 case. The patients who were considered to be abnormal or borderline were referred to the pediatric rheumatology unit, where 8 were diagnosed with osteoporosis (1 with hypophosphataemic rickets and salt-losing tubulopathy) and 4 were treated with alendronate.

Fundoscopy examinations were performed in 28 patients, and the results were abnormal in 4. Two presented systemic-hypertension-related lesions, 1 presented with a toxoplasmosis scar and 1 developed a cataract 1 year after the termination of pulse therapy. Urinary tract ultrasounds were performed in 37 patients and were found to be abnormal in 7 patients. Two presented with nephrolithiasis, 4 with nephrocalcinosis and 1 with a small pyelocaliceal dilatation.

None of the patients were diagnosed with diabetes mellitus. Three were referred to the pediatric endocrinology unit due to multiple hyperglycemic episodes. Each of these patients had normal levels of fasting plasma glucose and glycosylated hemoglobin.

Sixteen patients were diagnosed with systemic hypertension, but 9 of these were diagnosed before beginning pulse therapy.

## Discussion

Bronchiolitis obliterans has a number of causes, including connective tissue disorders, occupational inhalation injuries, hypersensitivity pneumonitis, drugs, radiation, Stevens-Johnson syndrome and lung, heart/lung or bone marrow transplantation [1,3,4].

In childhood, BO occurs most commonly after a severe lower respiratory tract infection and is considered a long-term sequela of viral infections [1,5,19]. Several agents have been associated with the development of post-infectious BO, such as adenovirus (types 3, 7 and 21), respiratory syncytial virus (RSV), influenza, parainfluenza, measles and *Mycoplasma pneumoniae* [5,10,20]. Adenovirus infection and the severity of acute illness (hospital days, intensive care unit admission, mechanical ventilation, oxygen use, corticosteroid treatment and β2-agonist administration) are the most significant risk factors for developing post-infectious BO in children [2,19,21]. This is consistent with our study, in which the presumed etiology was post-infectious in 37 patients, based on histories of prior infection and/or positive viral serology findings.

The diagnosis of BO was based on clinical criteria, findings on HRTC scans and/or open lung biopsies [7,10,22-25]. Although lung biopsy is the gold standard for diagnosis, the sensitivity is low due to the heterogeneous distribution of airway involvement throughout the lung parenchyma [1,3,10]. Lung biopsies are not usually needed when clinical conditions suggestive of post-infectious BO and specific HRCT scan abnormalities (mosaic perfusion, bronchiectasis) are present, once other possible causes of chronic obstructive pulmonary diseases have been excluded [1,7,22-24]. All 37 post-infectious BO patients in our study had histories of obstruction of the airways that persisted over 6 weeks after the initial event and had suggestive CT scans. Other diseases were excluded. Fourteen patients had open lung biopsies with pathologic findings consistent with obliterative bronchiolitis (2 with signs of aspiration).

Mauad et al. [26] studied the histopathological features of 34 pediatric patients with a diagnosis of BO and showed that childhood bronchiolitis obliterans is histologically characterized mainly by a constrictive pattern (97%), which is in agreement with the findings in our sample (13 constrictive patterns from 18 biopsies).

Pulmonary function tests in children with post-infectious BO typically show severe airway obstruction with small responses to bronchodilation, increased resistance, normal or increased total lung capacity (TLC) and elevated residual volume (RV) due to hyperinflation and air trapping [1,10,23,24]. In our study, all of the tested subjects had patterns of pulmonary function characterized by mild to severe obstruction and significantly diminished FEV_1_.

There is no consensus on to the optimal treatment for BO [6,8]. General supportive measures include avoidance of tobacco smoke and other inhaled irritants, the use of influenza vaccination, chest physiotherapy, adequate nutritional intake and the administration of supplemental oxygen for hypoxemic patients [1,10].

Although systemic corticosteroids are frequently used, their effectiveness in improving the outcomes for patients with BO, as well as the best route of administration, is controversial [1,23]. Some clinicians opt for the systemic use of corticosteroids rather than delivery by inhalation because the obliterative lesions in the small airways can interfere with the deposition of aerosol [8,23,25]. Others prefer inhaled corticosteroids to minimize the adverse systemic effects and to reduce airway hyperreactivity [6,19].

The advantages of intravenous pulse therapy are the enhancement of the therapeutic effect and reduction of the side effects caused by the intermittent administration of high doses of drugs [27-29]. Corticosteroid pulse therapy is arbitrarily defined as more than 250 mg prednisone or its equivalent per day [29]. The first reported use of a high-dose intravenous corticosteroid was in 1969, when it was used to successfully prevent renal allograft rejection [29]. Since then, high-dose intravenous corticosteroids have come to be used in a variety of inflammatory conditions [27-30].

In the field of pulmonology, pulse therapy has been effective in the treatment of other inflammatory lung diseases, such as chronic interstitial lung diseases [31,32]. There are limited reports of its use in patients with post-infectious BO [8]. Recently, it has been administered to pediatric patients with post-transplant BO. Ratjen et al. [33] studied nine children who were treated with pulse methylprednisolone therapy after being diagnosed with BO subsequent to bone marrow transplantation. The oxygen saturation in all of these individuals increased significantly and was normalized at the end of therapy. In 5 of the 9 patients, the treatment led to stabilization of the lung function without further deterioration during the follow-up period, but normalization of the pulmonary function was not achieved.

In our study, the children with BO who were treated with the high-dose methylprednisolone pulse therapy displayed clinical improvements as demonstrated by decreased wheezing exacerbations and improved oxygen saturation. The numbers of hospitalizations were reduced as a consequence. These data suggest that this therapy may be a valuable treatment option for children with BO.

Twenty-four months after the beginning of the pulse therapy, 83% of patients who received prolonged oral corticosteroid therapy were able to stop this prolonged therapy. Twenty-five percent of hypoxemic patients completely discontinued supplemental oxygen therapy, and in another 25% of patients, oxygen therapy was modified from full-time administration to overnight-only. Approximately 52% of the patients with evidence of pulmonary hypertension by echocardiogram exhibited normalization following the pulse therapy. In our opinion, the most important reason for recommending pulse therapy in patients with BO should be a dependence on oral corticosteroid therapy and a need for domiciliary oxygen therapy.

Comparing patients with and without prolonged oral corticosteroid therapy, we found that the difference in pulse therapy duration was not significantly different. The group with oral corticosteroids had a decrease in the number of hospitalizations but not of exacerbations, indicating that the exacerbation severity was reduced but not the frequency. The group without oral corticosteroids had a decrease in the number of exacerbations but not hospitalizations. The median hospitalization before the pulse therapy was already zero, indicating that exacerbations were less severe in this group.

With regards to safety, the toxicity of corticosteroids is manifested mainly by disturbances in electrolytes, suppressed growth, loss of bone mass, cushingoid appearance, gastrointestinal bleeding, neural complications, metabolic toxicity, renal complications, cardiac toxicity, ophthalmic problems and damage to the immunological system, which result in an increased susceptibility to infections [27-30,34,35]. In patients with normal kidney function, the renal effects of pulse therapy seem to be minimal. However, in nephritic patients they can lead to renal deterioration [36].

In our sample, all the acute effects during the methylprednisolone intravenous infusion were transient, and none were serious. Hyperglycemia occurred in 9% of cycles, hypernatremia in 1.7% and acute hypertension in 2.3%.

In our study, 8 patients presented with osteoporosis (1 had hypophosphatemic rachitis with phosphorus-losing tubulopathy), 1 developed a cataract one year after pulse therapy, 7 had abnormal urinary tract ultrasounds (2 cases of nephrolithiasis, 4 cases of nephrocalcinosis and 1 case of small pyelocaliceal dilatation), and 16 presented with systemic hypertension (9 of whom exhibited this condition prior to pulse therapy). None of the patients were diagnosed with diabetes mellitus.

Nevertheless, the adverse effects of corticosteroids are dose and time-dependent, so it is impossible to discriminate between prolonged corticosteroid therapy side effects due to pulse therapy and/or those due to any oral corticosteroid that the patients received for wheezing exacerbations. It is important to note that 23 patients were already on continuous oral corticosteroid therapy at the beginning of this treatment.

There is a general concern regarding the effect of corticosteroids in pediatric patients. However, in our study, the pulse therapy did not appear to have interfered with growth. Indeed, we observed improvements in height and weight, possibly because of the clinical stabilization. After pulse therapy, the mean Z-score of length by age improved from -1.08 to -0.63 and the mean Z-score of weight by age improved from -0.91 to -0.59.

As far as we know, this is the largest series of pediatric BO patients treated with methylprednisolone pulse therapy that has been reported to date. However, we are aware that the present study has a number of limitations. First, this study did not include a control group and therefore cannot definitively prove that treatment altered the natural course of the disease. Additionally, it is well known that the small airways are disproportionately narrow in the early years of life. However, lung growth increases peripheral airway conduction, which reduces airway resistance. The airways consequently become less vulnerable to obstruction [23]. Therefore, the clinical improvement observed in our sample of patients may be due to the physical increase in the sizes of the peripheral airways, which is the consequence of normal lung growth and does not necessarily represent regression of the pathology [19,23]. Finally, our cohort was relatively small because of the low frequency of this disease in pediatric patients. A multicenter approach would be needed to sample a larger patient cohort.

In contrast to the high mortality rate for post-transplantation BO (25-56%) [4,37,38], post-infectious BO tends to show clinical improvement after 2-3 years of supportive therapy, although clinical, radiological and spirometric changes might persist [8,19,23-25]. As shown in a study by Zhang et al. [23] of 31 patients with post-infectious BO, clinical remission was found in 22.6% of the patients, 67.7% had persistent respiratory signs and symptoms, and 9.7% of the patients died. In another study of 20 post-infectious BO patients [25], no improvement was evident in 40% of patients, 55% had partial remission of symptoms, 5% died of the disease and none recovered completely after 3 years of follow-up. In a Brazilian study of 48 patients with post-infectious BO [39], there was partial clinical improvement in 65% of patients, but 35% had no improvement at all after 3 years of follow-up.

However, the most worrisome results were found in the study by Cazzato et al. [40]. In this study, eleven patients with post-infectious BO were followed up for a mean period of 10.2 years, and FEV_1_ and FEF_25-75%_ fell at rates of -1.01% and -1.04% per year, respectively. These results suggest that childhood post-infectious BO can be a progressive lung disorder with increasing lung function impairment. Consequently, patients may need an anti-inflammatory treatment to prevent the progression of the lesions.

## Conclusions

In conclusion, our results suggest that intravenous methylprednisolone pulse therapy appears to be useful and fairly safe in pediatric patients with bronchiolitis obliterans. This treatment can modify the progress of the disease and may be an alternative option for administering systemic corticosteroids and thereby avoiding the side effects associated with continuous oral corticotherapy. Further prospective controlled studies are necessary to confirm these findings.

## Abbreviations

BO: Bronchiolitis obliterans
HRCT: High Resolution Computed Tomography of the lungs
FVC: Forced vital capacity
FEV_1_: Forced expiratory volume in one second
FEF_25-75%_: Forced expiratory flow between 25% and 75% of FVC
